# The Influence of Socio-Ecological Networks on Willingness to Communicate in English for Japanese People

**DOI:** 10.3389/fpsyg.2021.580448

**Published:** 2021-07-09

**Authors:** Takehiko Ito

**Affiliations:** Department of Information Networking for Innovation and Design, Toyo University, Tokyo, Japan

**Keywords:** network analysis, socioecology, second language, willingness to communicate, relational mobility

## Abstract

This study investigates the effect of socio-ecological networks on the willingness to communicate (WTC) in English among Japanese people. Previous studies have shown that relational mobility (socio-ecological factor), which is defined as the availability of opportunities to choose new relationship partners, positively affects the WTC in English for Japanese people. However, the network structure of the variables of relational mobility and its effects have not been revealed yet. The present study conducted network analysis with 474 Japanese university students and found the two clusters that correspond to the dimensions of relational mobility in the partial correlation network. Three variables regarding opportunities to meet new people and leave current relationships positively affected the WTC in English; one had a negative effect. Centrality indices, such as nodes strength, betweenness, and closeness, revealed the centrality of several variables in the network. Bootstrapping methods showed the trustworthiness of the estimated network structure and centrality indices as well as edges and variables whose effects differed significantly from that of others. Contrary to the regression analysis results, the network analysis findings can help us understand the in-depth effect of relational mobility on the WTC in a second language, which will prove useful for intervention studies.

## Introduction

Researchers must identify the factors influencing attitudes toward second language communication to be able to promote positivity among language learners. Many studies have addressed the psychological processes shaping communication attitudes toward second languages using Canadians as participants (MacIntyre and Charos, 1996; MacIntyre et al., 1998). There have also been studies among Asian peoples, such as the Japanese (Yashima et al., 2004, 2018; Ito, 2013), Chinese (Peng and Woodrow, 2010), Koreans (Li, 1998), and Malaysians (Yousef et al., 2013). Researchers have proposed the willingness to communicate (WTC) signals a positive attitude toward language communication (MacIntyre et al., 1998). WTC in a second language is defined as readiness to enter into a discussion voluntarily at a specific time with one or more people using a second language (MacIntyre et al., 1998). Positive correlations between WTC and frequency of communication in a second language have been reported (MacIntyre and Charos, 1996; Yashima et al., 2004). Research has mainly focused on internal factors, such as confidence in language communication and Big Five personality traits, to predict WTC, and neglected the influence of social structures (Gallagher, 2019).

According to Peng (2012), EFL students’ classroom WTC is socially constructed as a function of the interaction of individual and environmental factors. She found that the classroom atmosphere influenced the participants’ communication behavior considerably, both positively and negatively. Peng (2014) offered the socio-ecological framework for WTC and, using Chinese university students, showed that WTC in the classroom was influenced by a number of factors: classroom atmosphere, teacher factors, interlocutors and communicative situations, group mates’ participation, and tasks. She stated that a learners’ cognitive, linguistic, and affective conditions were embedded in the classroom, and the individual and environmental factors influenced their WTC.

Targeting Chinese students studying abroad in New Zealand, Cao (2014) showed that WTC was interpreted as a dynamic situational variable rather than a trait disposition where WTC was influenced by individual characteristics, classroom environmental conditions, and linguistic factors. Peng and Woodrow (2010) targeted Chinese university students in the eastern area of China and determined that teacher support, student cohesiveness, task orientation (defined as classroom environment), and communication confidence in English positively predicted WTC in English.

These socio-ecological frameworks for WTC mainly focused on the classroom. In the classroom, students communicate with teachers and other classmates. However, university students communicate with not only each other, but also workplace colleagues and people from various communities. Therefore, the present study used the socio-ecological framework, focusing on an individual’s social surroundings, and relational mobility was used as the socio-ecological factor.

To predict WTC, the present study focused on relational mobility, which is the degree to which individuals in a given social context perceive that they can form and terminate interpersonal relationships and groups (Yuki et al., 2007; Schug et al., 2010; Thomson et al., 2018). It measures the societal features of opportunities to meet new people and choose interactional partners and groups (Schug et al., 2009). Relational mobility is an individual’s surrounding environment, such as friends’, school mates’, and colleagues’ relational mobility.

People in Singapore and Taiwan do not have many chances to meet new people or the freedom to form and terminate interpersonal relationships, which means a lower tendency of relational stability (Wang et al., 2011). People in a society with higher relational mobility tend to form more new interpersonal relationships (Schug et al., 2010). According to Yamagishi (1998), interpersonal relationships and social networks are less flexible in Japan than in American society. Japanese people have fewer opportunities to build new relationships and tend not to interact positively with people outside their groups. A lower tendency of WTC in English for Japanese people may be caused by the low relational mobility of the society. Relational mobility appears to influence the English communication attitudes of Japanese people; Ito (2019) performed regression analysis and found that relational mobility positively influenced WTC in English for Japanese people. Furthermore, according to Ito (2021), relational mobility positively influenced WTC in English via perceived competence in English for Japanese people.

However, previous studies have not shown the network structure of the variables of relational mobility and its effect on WTC. There is a gap in the literature on how the variables relate to each other, how much influence each holds, and what the central factor is. Since relational mobility is related to WTC, it is important to examine the effect of each variable of relational mobility on WTC for specific intervention studies, where a bundle of variables of relational mobility cannot be suggested.

This study conducted network analysis to examine the partial correlation network structure of relational mobility. Network analysis allows for multiple factors to be considered (Borgatti et al., 2009). In psychiatry, the network approach is gaining popularity. The position of the nodes in the network is based on an algorithm, which makes strongly correlated symptoms cluster in the middle; symptoms with weaker connections to other symptoms go to the periphery (Fruchterman and Reingold, 1991). Analyzing symptoms as a network makes it possible to examine how symptoms associate and relate to each other (Cramer et al., 2010).

Latent variable models show shared variance among symptoms, whereas network analyses estimate unique variance between symptoms. Network models can suggest causal relationships among symptoms. Latent variable models can only show that an underlying common factor is causing multiple symptoms, accounting for symptom covariation (Smith et al., 2018). Compared with structural equation modeling (SEM) and mediation analysis (MA), network analysis can use a greater number of variables at once. SEM and MA are more appropriate for a smaller number of variables, especially if there is a clear theoretical expectation for the relationships between the variables (Letina et al., 2019).

## Materials and Methods

### Participants

The participants were 469 Japanese undergraduate students from four universities in Tokyo and Kanagawa prefectures (163 men, 302 women, 4 others; mean age = 19.69 years, *SD* = 1.12). The rankings of these universities in Japan were around average, and the participants’ English proficiency levels were also around average. Their majors were computer science, psychology, art and design, and marine biology. Every student takes an English speaking and listening class and an English reading and writing class in the first year. These compulsory courses are taught by a native English speaker or a Japanese English speaker, using standard English textbooks published by Japanese companies. The participants were from four universities, and they also belonged to different circles and communities, did part-time work, and lived in different areas. Therefore, the relational mobility in the present study did not intend to determine how the participants perceived the same environment differently.

### Questionnaire

#### Relational Mobility

The relational mobility scale was published by Yuki et al. (2007), and the Japanese version was used in the present study. The participants answered each item based on the overall question, “How much do each of the following statements accurately describe the people in the immediate society (your school, workplace, town, neighborhood, etc.) in which you live?” This scale consisted of two dimensions. The items concerning meeting new people included: “(1) They have many chances to get to know other people”; “(2) It is common for these people to have a conversation with someone they have never met before”; “(3) There are few opportunities for these people to form new friendships”; “(4) It is uncommon for these people to have a conversation with people they have never met before”; and “(5) It is easy for them to meet new people.” The items concerning the choice of one’s own interaction partners included: “(6) They can choose who they interact with”; “(7) If they did not like their current groups, they would leave for better ones”; “(8) It is often the case that they cannot freely choose who they associate with”; “(9) Even if these people were not completely satisfied with the group they belonged to, they would usually stay with it anyway”; “(10) These people can choose the groups and organizations they belong to”; “(11) Even if these people were not satisfied with their current relationships, they would often have no choice but to stay with them”; and “(12) Even though they might rather leave, these people often have no choice but to stay in groups they don’t like.” Items 3, 4, 8, 9, 11, and 12 were reversed. The number of each item corresponds to its node in the network analysis results. Twelve items were used for relational mobility (α = 0.78). The response options for all statements ranged from 1 (not at all) to 5 (very).

#### WTC in English

The WTC scale was published by McCroskey (1992), and the Japanese version (Yashima et al., 2004) was used in the present study. The participants answered how willing they were to perform what each item described. This scale consisted of four communication contexts (talking in dyads, small groups, large meetings, and in front of an audience) with three types of receivers: strangers, acquaintances, and friends. Twelve items were used for WTC in English (α = 0.95, acquaintances: “Talking with an acquaintance,” “Talking with a small group of acquaintances,” “Talking in a large meeting of acquaintances,” “Presenting a talk to a group of acquaintances”; strangers: “Talking with a stranger,” “Talking with a small group of strangers,” “Talking in a large meeting of strangers,” “Presenting a talk to a group of strangers”; and friends: “Talking with a friend,” “Talking with a small group of friends,” “Talking in a large meeting of friends,” and “Presenting a talk to a group of friends”). The response options for all statements ranged from 1 (not at all) to 5 (very).

### Procedure

Questionnaires with scales assessing relational mobility and WTC in English were administered to students in the classes. The instructors distributed paper-based questionnaires or links to web-based questionnaires for the students to complete. The first page of the questionnaire contained information written in Japanese that the students’ participation was voluntary and anonymous. The students provided their consent to participate.

## Results

**FIGURE 1:**
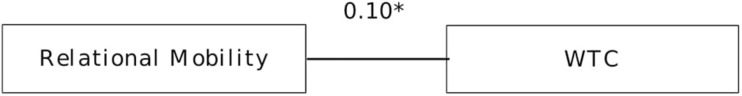
The regression analysis results of relational mobility on WTC in English. *p < 0.05.

Before conducting the network analysis, we examined whether relational mobility positively influenced WTC in English. The regression analysis of relational mobility on WTC in English revealed a significant positive effect (β = 0.10, *p* < 0.05; Figure 1), replicating the results found by Ito (2019).

After we checked the replication, to examine the partial correlation network structure of relational mobility and its effect on WTC in English, we used LASSO (least absolute shrinkage and selection operator) regularization with EBIC (minimizing the extended Bayesian information criterion) model selection. The LASSO shrinks partial correlation coefficients for estimating a network structure whose edges show the value of association between nodes, after controlling the influence of all other nodes in the network, and sets small coefficients to zero (Epskamp, 2017). EBIC was used to find the true network structure (Foygel Barber and Drton, 2015; Foygel and Drton, 2010). Therefore, LASSO regularization with EBIC does not estimate edges that are not in the true network; it estimates edges that are in the true network based on the true network structure and sample size (Epskamp, 2017). LASSO needs considerable power to get stable parameter estimates (Epskamp, 2017).

**FIGURE 2:**
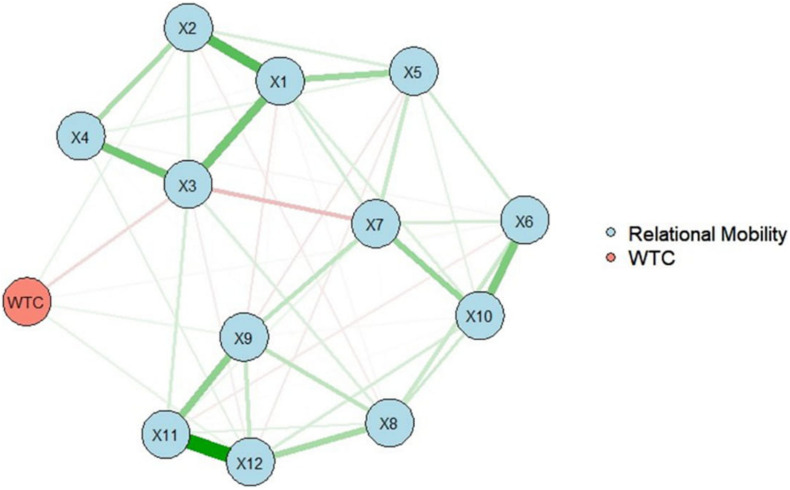
The network structure of relational mobility and WTC in English. Nodes represent observed variables, and links represent partial correlations between two variables, after controlling for all other variables. Node numbers correspond to the variable numbers in “Materials and Methods” section.

For the analysis, the R package *qgraph* (Epskamp et al., 2012) and *glasso* (Friedman et al., 2014) were used to estimate a partial correlation network using EBIC selection (Figure 2). The scale of relational mobility has the two dimensions “meeting new people” (variables 1–5) and “choice of one’s own interaction partners” (variables 6–12). Variables 3, 4, 8, 9, 11, and 12 were reversed. As a result, the variables of relational mobility had the two clusters of the dimensions in the network. Therefore, the network structure shows the two dimensions well. Furthermore, four variables of relational mobility directly affected WTC in English: Variables 2, “It is common for these people to have a conversation with someone they have never met before,” 9, “Even if these people were not completely satisfied with the group they belonged to, they would usually stay with it anyway,” and 12, “Even though they might rather leave, these people often have no choice but to stay in groups they don’t like” had positive effects; variable 3, “There are few opportunities for these people to form new friendships” had a negative effect.

In addition, the network structure was revealed and node centrality was examined. The importance of each node in the network is indicated by the node centrality. Node strength sums up the strength of all connected edges to a node. The network is the partial correlation coefficients, and the node strength shows the sum of absolute partial correlation coefficients between the node and all other nodes. Closeness indicates the inverse of the sum of all the shortest paths between the node and all other nodes in the network. Betweenness refers to how many of the shortest paths between two nodes go through the node; the higher the betweenness, the more important the node is in connecting other nodes (Epskamp, 2017).

**FIGURE 3:**
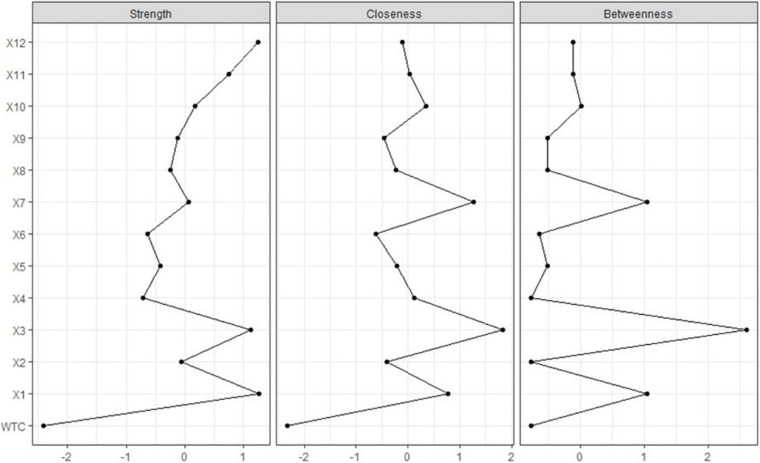
Centrality indices are shown as standardized z-scores.

For the analysis, the function *centralityPlot* was used to estimate the centrality of the partial correlation network using EBIC selection (Foygel and Drton, 2010; Figure 3). For strength, variables 1, “They have many chances to get to know other people,” 3, “There are few opportunities for these people to form new friendships,” and 12, “Even though they might rather leave, these people often have no choice but to stay in groups they don’t like” exceeded one. For closeness, variables 3 and 7, “If they did not like their current groups, they would leave for better ones” exceeded one. For betweenness, variables 1, 3, and 7 exceeded one. The centrality indices of almost all the variables were around or under zero. Therefore, the variables over 1 mean high centrality.

Were these results replicable? To overcome replication concern, bootstrapping methods were used. The *bootnet* package can estimate different network models and assess the accuracy of the estimated network structure (Epskamp, 2017). The package includes the bootstrapping methods, *CS*-coefficient, and bootstrapped difference tests. After estimating nonparametric bootstraps, *bootnet* returns plots that show the bootstrapped CIs of edge-weights, which refers to which edges and nodes significantly differ from one another (Epskamp, 2017).

**FIGURE 4:**
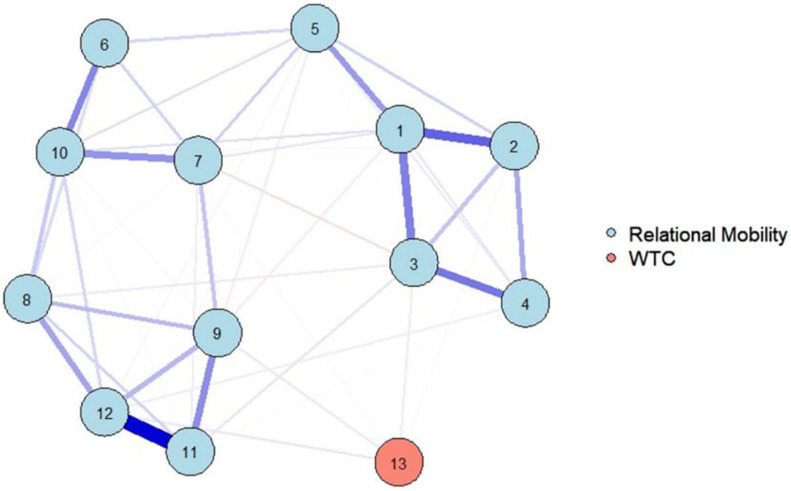
The estimated network structure of relational mobility and WTC by bootstrapping methods. The network structure is a Gaussian graphical model, which is a network of partial correlation coefficients. Node numbers correspond to the variable numbers in “Materials and Methods” section, and No. 13 refers to WTC.

For the analysis, the function *estimateNetwork* was used to estimate the network structure (Figure 4). As in Figure 2, the variables of relational mobility had the two clusters of the dimensions in the network. Therefore, the network structure shows the two dimensions well. Furthermore, three variables of relational mobility directly affected WTC in English: variables 9 and 12 had positive effects, and variable 3 had a negative effect. Contrary to Figure 2, variable 2 did not directly affect WTC in English.

**FIGURE 5:**
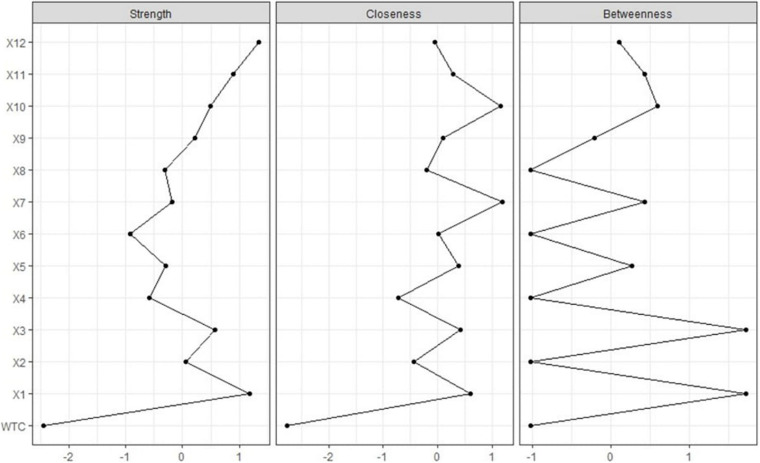
Centrality indices are shown as standardized z-scores.

Furthermore, the function *centralityPlot* was used to estimate the centrality of the partial correlation network using EBIC selection (Foygel and Drton, 2010; Figure 5). For strength, variables 1 and 12 exceeded one; for closeness, variables 7 and 10, “These people can choose the groups and organizations they belong to” exceeded one; and for betweenness, variables 1 and 3 exceeded one. This centrality estimation nearly overlaps with that of Figure 3.

**FIGURE 6:**
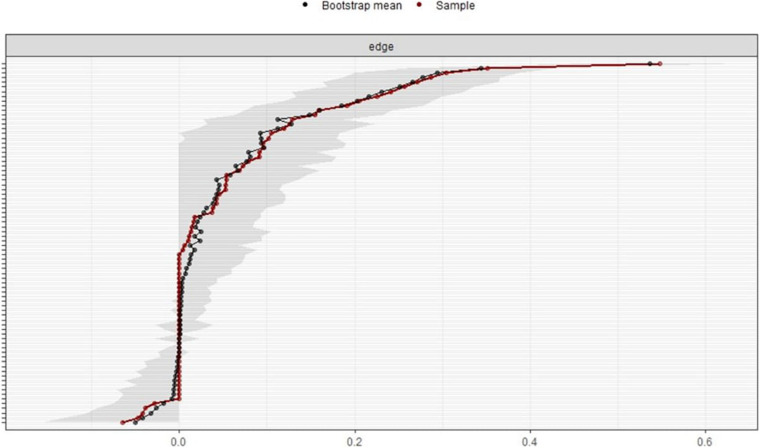
Bootstrapped confidence intervals of estimated edge-weights for the estimated network. The red line shows the sample values, and the gray area shows the bootstrapped CIs. Each horizontal line represents one edge of the network. The top is the highest edge-weight, and the bottom is the lowest edge-weight. Y-axis labels were removed to avoid cluttering.

Figure 6 depicts bootstrapped CIs around the estimated edge-weights, indicating that many edge-weights likely do not differ significantly from one another. The bootstrapped CIs imply that interpreting the order of most edges in the network should be done with care (Epskamp, 2017). The single strongest edge between variables 11 and 12 is reliable as their bootstrapped CIs do not overlap with the bootstrapped CIs of any other edges. Non-overlapping CIs indicate two statistics significantly differ at the given significance level (Epskamp, 2017). In the *bootnet* function, the *nBoots* (*n* = 2,500, this time) argument was used to achieve smoother plots. The *nCores* (*n* = 8, this time) argument was used to speed up bootstrapping and to use multiple computer cores. The *plot* function was used to plot the bootstrapped CIs for estimates.

**FIGURE 7:**
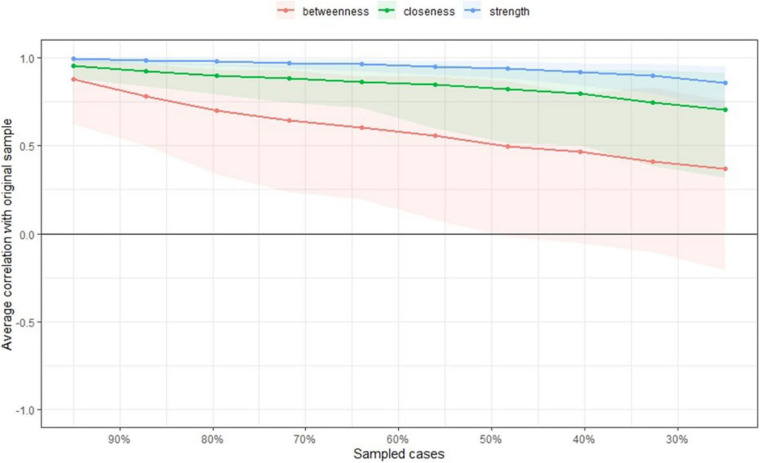
Average correlations between the centrality indices of networks sampled with participants dropped and the original participants. Colored lines depict the means, and areas depict the range from the 2.5th quantile to the 97.5th quantile.

Next, we examined the stability of the centrality indices of the estimated network models based on subsets of the data. The case-dropping bootstrap by the *corStability* function was used. Figure 7 shows the resulting plot: the stability of betweenness dropped steeply, while the stability of node strength and closeness improved. *CS*-coefficient calculates the maximum proportion of cases that can be dropped to retain a correlation with the original centrality of higher than (by default) 0.7 (Epskamp, 2017). The *CS-*coefficient of betweenness was not good [*CS*(cor = 0.7) = 0]. Node strength performed better [*CS*(cor = 0.7) = 0.75], as did closeness [*CS*(cor = 0.7) = 0.28].

**FIGURE 8:**
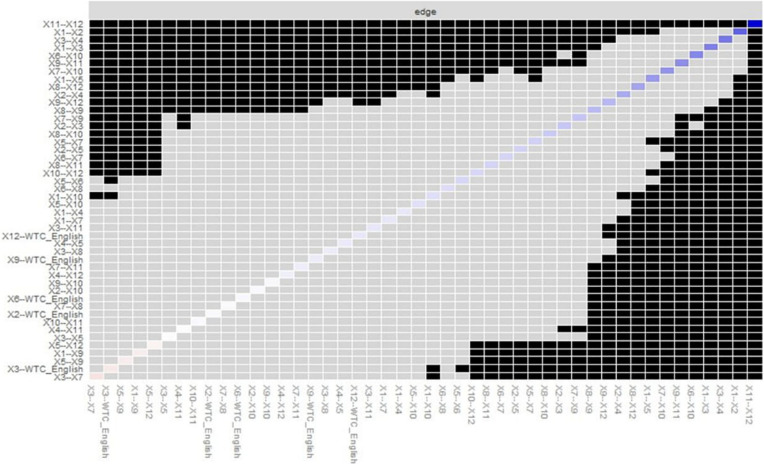
Bootstrapped differences (α = 0.05) between edge-weights that were non-zero in the estimated network. Gray boxes indicate edges that do not differ significantly from one another. Black boxes indicate edges that differ significantly from one another. Colored boxes correspond to the colors of the edges in Figure 4.

Finally, the *differenceTest* function was used to compare edge-weights with the bootstrapped difference test. It uses non-parametric bootstrap results rather than case-dropping bootstrap results. The *plot* function was used to plot the difference tests between all pairs of edges (Figure 8). The *plot* argument was used because the function plots bootstrapped CIs for edge-weights. The *onlyNonZero* argument was used so that the only edges shown are nonzero in the estimated network; the order argument orders the edge-weights from the most positive to the most negative edge-weight in the sample network (Epskamp, 2017). According to Figure 8, the edge-weight between variable 11, “Even if these people were not satisfied with their current relationships, they would often have no choice but to stay with them,” and variable 12 differed significantly from all other edge-weights.

**FIGURE 9:**
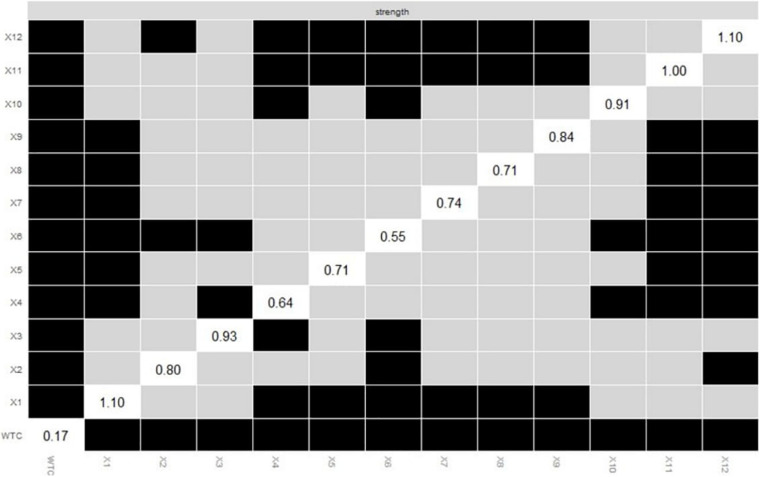
Bootstrapped differences (α = 0.05) between the node strengths of the thirteen variables. Gray boxes indicate nodes that do not differ significantly from one another. Black boxes indicate nodes that differ significantly from one another. White boxes in the centrality plot show the value of the node strength.

The *plot* function was also used to plot the differences of the centrality indices between all nodes. According to Figure 9, the node strength of variable 12 showed more differences than all the other nodes.

## Discussion

Before conducting the network analysis, the regression analysis of relational mobility on WTC in English revealed a significant positive effect, reproducing the findings from Ito (2019). The socio-ecological factor positively influenced WTC in English for Japanese people.

For revealing the in-depth effect of relational mobility on WTC by examining the partial correlation network structure of relational mobility and its effect on WTC, LASSO regularization with EBIC model selection was used. The two clusters of the network structure of relational mobility, which were “meeting new people” and “choice of one’s own interaction partners,” were accurately located. Four variables directly affected WTC in English: Variables 2, 9, and 12 had positive effects, whereas variable 3 had a negative effect. Although regression analysis showed that relational mobility positively influenced WTC in English, network analysis showed variable 3 had a negative effect on it. This result suggests that, after controlling for all other variables, some variables of relational mobility did not impact WTC in English; in fact, one had a negative effect.

After showing the network structure, it is important to examine the centrality. The importance of each node in the network was indicated by the node centrality. For strength, variables 1, 3, and 12 exceeded one; for closeness, variables 3 and 7 exceeded one; and for in betweenness, variables 1, 3, and 7 exceeded one. Variable 3, “There are few opportunities for these people to form new friendships” exceeded one in the three indices, meaning it could be a central variable in the network, even though its impact on WTC in English is negative. The result suggests that if the central variables change, the others will change simultaneously. Therefore, if we want to change WTC in English, changing these central variables will be effective because these variables change the structure of relational mobility largely, and the changed relational mobility will influence WTC. Regression analysis cannot find central variables in the network and tell which variables effectively change a bundle of variables of relational mobility. However, it is important to check whether the network result is replicable.

To overcome replication concern, bootstrapping methods were used on the network structure with the same two clusters as that used in the LASSO regularization with EBIC model selection. Therefore, the structure was replicated with bootstrapping methods. Three variables of relational mobility directly affected WTC in English: Variables 9 and 12 had positive effects, and variable 3 had a negative effect. Variable 2 did not affect WTC. These results showed that the network estimation of the LASSO regularization was trustworthy.

Furthermore, the centrality indices of the estimated network with bootstrapping methods showed that for strength, variables 1 and 12 exceeded one; for closeness, variables 7 and 10 exceeded one; and for betweenness, variables 1 and 3 exceeded one. This centrality estimation almost overlapped with the original network, showing the trustworthiness of its centrality indices.

Bootstrapped CIs around the estimated edge-weights showed that many edge-weights likely did not differ significantly from one another. The strongest edge occurred between variables 11 and 12 whose bootstrapped CIs did not overlap with the bootstrapped CIs of any other edges. The edge had the strongest value in the network.

Then, the centrality indices of the estimated network models, based on data subsets, showed that the stability of betweenness dropped steeply, while that of node strength and closeness were better. The *CS*-coefficient of betweenness was not good, while node strength and closeness performed better. Therefore, the orders of node strength and closeness may be interpretable, while the orders of betweenness are not.

Lastly, comparing edge-weights using the bootstrapped difference test showed that the edge-weight between variables 11 and 12 differed significantly from all other edge-weights. Comparing nodes using the bootstrapped difference test revealed that the node strength of variable 12 was the most different from all other nodes. These results suggest that variable 12 worked more differently than did the other variables in the network. As the centrality indices of strength indicated, variable 12 had high centrality, and this variable had the power to change the relational mobility as a whole and WTC, which regression analysis cannot suggest.

Previous studies have not reported on the network structure of the variables of relational mobility and its effect on human attitudes. In this study, network analysis showed that the partial correlation network had the two clusters that correspond to the dimensions of relational mobility. Some variables positively influenced WTC in English, but one discouraged it. Several variables showed centrality in the network structure. Furthermore, bootstrapping methods showed the trustworthiness of the estimated network structure and centrality indices as well as edges and variables that had significantly different effects than all the others. These findings are useful for intervention studies in language education because they suggest an effective way to change the socio-ecological factors and WTC.

One limitation of this study is that it did not focus on other nationalities, such as an American population. According to Yamagishi (1998), interpersonal relationships and social networks are flexible in western countries, where individuals have opportunities to build new relationships and tend to trust others in general. Based on these findings, we expect the relational mobility of a western population to be high, and its partial correlation network structure and effects on WTC to differ from that of the present study. Future research should focus on the relational mobility of multiple countries to generalize the findings of this study and compare them to make the findings convincing.

The present study focused on relational mobility, which was broken up into items, to compare the previous analyses, such as regression analysis. However, if WTC is broken up into the different contexts (“dyads,” “small groups,” “large meetings,” and “audience”) or different receivers (“strangers,” “acquaintances,” and “friends”), the analysis reveals the whole structure of the psychological network. A future study will examine the subscales of WTC in the network.

Even though students who attend the same university have different work or living environments, these environments are partially overlapped, that is to say, at university. The present study did not compare students who had partially overlapped environments with those who had completely different environments. Relational mobility in the present study did not indicate how the participants perceived the same environment differently; however, to make this point convincing, future studies should investigate the effect of partially overlapped environments.

## Conclusion

The present study showed the network structure of the variables of relational mobility and its effect on WTC in English for Japanese people. Contrary to the regression analysis results, the findings from our network analysis showed how each variable related to each other and which variable was central. Even though previous studies have shown the effect of socio-ecological factors on WTC, the present study revealed the network structure of socio-ecological factors and how the variables influenced WTC and each other simultaneously. Therefore, WTC can be interpreted as a dynamic situational variable.

These findings can help us understand the in-depth effect of relational mobility on WTC in a second language; an implication lacking from the regression analysis of a bundle of variables. Through the network analysis, we now know which variables of relational mobility effectively change the relational mobility and, consequently, change WTC. Therefore, it helps us prepare intervention studies by showing specific variables that efficiently affect WTC in a second language.

## Data Availability Statement

The raw data supporting the conclusions of this article will be made available by the authors, without undue reservation.

## Ethics Statement

The studies involving human participants were reviewed and approved by the Research Committee of the Center for English as a Lingua Franca at Tamagawa University, Japan (ID: 151149). The patients/participants provided their written informed consent to participate in this study.

## Author Contributions

The author made substantial contributions to design and acquisition of data, and analysis and interpretation of data. TI drafted the article for important intellectual content, and gives final approval of the version to be submitted.

## Conflict of Interest

The author declares that the research was conducted in the absence of any commercial or financial relationships that could be construed as a potential conflict of interest.

## References

[B1] Borgatti S. P. Mehra A. Brass D. J. Labianca G. (2009). Network analysis in the social sciences. *Science* 323 892–895. 10.1126/science.1165821 19213908

[B2] Cao Y. (2014). A sociocognitive perspective on second language classroom willingness to communicate. *TESOL Quart.* 48 789–814. 10.1002/tesq.155

[B3] Cramer A. O. J. Waldorp L. J. van der Maas H. L. J. Borsboom D. (2010). Comorbidity: a network perspective. *Behav. Brain Sci.* 33 137–150. 10.1017/S0140525X09991567 20584369

[B4] Epskamp S. (2017). *Network Psychometrics Ph, D.* Amsterdam: University of Amsterdam.

[B5] Epskamp S. Cramer A. Waldorp L. Schmittmann V. D. Borsboom D. (2012). qgraph: network visualizations of relationships in psychometric data. *J. Statist. Soft.* 48 1–18. 10.18637/jss.v048.i04

[B6] Foygel R. Drton M. (2010). Extended bayesian information criteria for gaussian graphical models. *Adv. Neural Inform. Proc. Syst.* 23 2020–2028.

[B7] Foygel Barber R. Drton M. (2015). High-dimensional ising model selection with bayesian information criteria. *Electron. J. Statist.* 9 567–607. 10.1214/15-EJS1012

[B8] Friedman J. H. Hastie T. Tibshirani R. (2014). *glasso: Graphical Lasso Estimation of Gaussian Graphical Models [Computer Software Manual].* Available online at: https://CRAN.R-project.org/package=glasso (accessed July 6, 2020).

[B9] Fruchterman T. Reingold E. (1991). Graph drawing by force-directed placement. *Soft. Pract. Exp.* 21 1129–1164. 10.1002/spe.4380211102

[B10] Gallagher H. C. (2019). Social networks and the willingness to communicate: reciprocity and brokerage. *J. Langu. Soc. Psychol.* 38 194–214. 10.1177/0261927X18809146

[B11] Ito T. (2013). Psychological processes predicting the English communication behavior of Japanese high school students. *Japan. J. Psychol.* 84 488–497. 10.4992/jjpsy.84.488 24505975

[B12] Ito T. (2019). “The effect of relational mobility on English communication attitude,” in *Proceeding of the Paper presented at the 60th Congress of the Japanese Society of Social Psychology*, (Tokyo).

[B13] Ito T. (2021). The influence of relational mobility on the willingness to communicate in English of Japanese people. *Japan. J. Soc. Psychol.* 37 15–25.

[B14] Letina S. Blanken T. F. Deserno M. K. Borsboom D. (2019). Expanding network analysis tools in psychological networks: Minimal spanning trees, participation coefficients, and motif analysis applied to a network of 26 psychological attributes. *Complexity* 2019 1–27. 10.1155/2019/9424605

[B15] Li D. (1998). It’s always more difficult than you plan and imagine: teachers’ perceived difficulties in introducing the communicative approach in South Korea. *TESOL Quart.* 32 677–703. 10.2307/3588000

[B16] MacIntyre P. D. Charos C. (1996). Personality, attitudes, and affect as predictors of second language communication. *J. Lang. Soc. Psychol.* 15 3–26. 10.1177/0261927X960151001

[B17] MacIntyre P. D. Clément R. Dörnyei Z. Noels K. A. (1998). Conceptualizing willingness to communicate in a L2: a situational model of L2 confidence and affiliation. *Mod. Lang. J.* 82 545–562. 10.1111/j.1540-4781.1998.tb05543.x

[B18] McCroskey J. C. (1992). Reliability and validity of the willingness to communicate scale. *Commun. Quart.* 40 16–25. 10.1080/01463379209369817

[B19] Peng J. (2012). Towards an ecological understanding of willingness to communicate in EFL classrooms in China. *System* 40 203–213. 10.1016/j.system.2012.02.002

[B20] Peng J. (2014). *Willingness to Communicate in the Chinese EFL University Classroom: An Ecological Perspective.* Bristol: Multilingual Matters.

[B21] Peng J. E. Woodrow L. (2010). Willingness to communicate in English: a model in the Chinese EFL classroom context. *Lang. Learn.* 60 834–876. 10.1111/j.1467-9922.2010.00576.x

[B22] Schug J. Yuki M. Horikawa H. Takemura K. (2009). Similarity attraction and actually selecting similar others: how cross-societal differences in relational mobility affect interpersonal similarity in Japan and the USA Asian. *J. Soc. Psychol.* 12 95–103. 10.1111/j.1467-839X.2009.01277.x

[B23] Schug J. Yuki M. Maddux W. W. (2010). Relational mobility explains between- and within-culture differences in self-disclosure toward close friends. *Psychol. Sci.* 21 1471–1478. 10.1177/0956797610382786 20817913

[B24] Smith K. E. Crosby R. D. Wonderlich S. A. Forbush K. T. Mason T. B. Moessner M. (2018). Network analysis: an innovative framework for understanding eating disorder psychopathology. *Int. J. Eating Dis.* 51 214–222. 10.1002/eat.22836 29451959 PMC5946321

[B25] Thomson R. Yuki M. Talhelm T. Schug J. Kito M. Ayanian A. H. (2018). Relational mobility predicts social behaviors in 39 countries and is tied to historical farming and threat. *Proc. Natl. Acad. Sci. U.S.A.* 115 7521–7526. 10.1073/pnas.1713191115 29959208 PMC6055178

[B26] Wang C. S. Leung A. K. Y. See Y. H. M. Gao X. Y. (2011). The effects of culture and friendship on rewarding honesty and punishing deception. *J. Exp. Soc. Psychol.* 47 1295–1299. 10.1016/j.jesp.2011.04.011

[B27] Yamagishi T. (1998). *The Structure of Trust: The Evolutionary Game of Mind and Society.* Tokyo: University of Tokyo Press.

[B28] Yashima T. MacIntyre P. Ikeda M. (2018). Situated willingness to communicate in an L2: interplay of individual characteristics and context. *Lang. Teach. Res.* 22 115–137. 10.1177/1362168816657851

[B29] Yashima T. Nishide L. Shimizu K. (2004). The influence of attitudes and affect on willingness to communicate and second language communication. *Lang. Learn.* 54 119–152. 10.1111/j.1467-9922.2004.00250.x

[B30] Yousef R. Jamil H. Razak N. (2013). Willingness to communicate in English: a study of malaysian pre-service English teachers. *Eng. Lang. Teach.* 6 205–216. 10.5539/elt.v6n9p205

[B31] Yuki M. Schug J. Horikawa H. Takemura K. Sato K. Yokota K. (2007). “Development of a scale to measure perceptions of relational mobility in society,” in *Proceeding of the CERSS Working Paper 75, Center for Experimental Research in Social Sciences*, (Hokkaido University).

